# Epibiotic macrofauna on common minke whales, *Balaenoptera acutorostrata* Lacépède, 1804, in Icelandic waters

**DOI:** 10.1186/1756-3305-6-105

**Published:** 2013-04-17

**Authors:** Droplaug Ólafsdóttir, Andrew P Shinn

**Affiliations:** 1 Marine Research Institute, Skulagata 4, Reykjavik IS 101, Iceland; 2 Institute of Aquaculture, University of Stirling, Stirling FK9 4LA, UK; 3 Present address: Icelandic Meteorological Office, Bústaðavegur 7-9 150-, Reykjavik, Iceland

**Keywords:** *Caligus elongatus*, *Pennella balaenopterae*, *Cyamus balaenopterae*, *Conchoderma* spp, *Xenobalanus globicipitis*, *Udonella caligorum*, *Petromyzon marinus*

## Abstract

**Background:**

Whilst there is a body of scientific literature relating to the epibiotic macrofauna on large whales, there is little information on the cetaceans in Icelandic waters. Common minke whales, *Balaenoptera acutorostrata* Lacépède, 1804, are a common sighting between the months of April to November, however, the migration and distribution of the population in winter requires establishing. The present study provides baseline information on the species composition, geographic distribution and abundance of the epibiotic macrofauna on minke whales landed in Icelandic waters and comments on their acquisition.

**Methods:**

The epibiotic macrofauna and skin lesions on 185 and 188 common minke whales respectively, landed in Icelandic waters between April to September 2003-2007 were determined. For each whale, the fluke and one lateral side was examined.

**Results:**

A total of seven epibiotic species were found: the caligid copepod *Caligus elongatus* (prevalence (P) = 11.9%, mean intensity (M.I) = 95.5); the pennellid copepod *Pennella balaenopterae* (P = 10.3%, M.I = 1.6); the cyamid amphipod *Cyamus balaenopterae* (P = 6.5%, M.I = 37.0); the lepadid cirripedes *Conchoderma virgatum* (P = 0.5%, M.I = 4.0) and *Conchoderma auritum* (P = 0.5%, M.I = 1.0), the balanid cirriped *Xenobalanus globicipitis* (P = 1.6%, M.I = 5.3) and the sea lamprey *Petromyzon marinus* (P = 2.7%, M.I = 1.0). In addition, the hyperparasitic monogenean *Udonella caligorum* was found on *C. elongatus* (P = 6.6%) on 8 of the 22 whales infected with the copepod. No significant relationship was observed between parasite intensity and host body length for either *C. balaenopterae* or *C. elongatus,* while the proportion of infected hosts was higher in August-September than earlier in the summer for *C. balaenopterae* (*χ*2 = 13.69; p<0.01: d.f.=1) and *C. elongatus* (*χ*2 = 28.88; p<0.01: d.f.=1).

**Conclusions:**

The higher prevalence of *C. balaenopterae* on male whales (*χ*2 = 5.08; p<0.05: d.f.=1), suggests possible different migration routes by the sexes. A likely explanation of the occurrence of *P. marinus* attached to the minke whales may be due to the gradually rising sea temperature in the area in recent years. This study represents the first known record of *C. elongatus* on a cetacean host.

## Background

Information on the epibiotic macrofauna on large whales has been reviewed repeatedly in the cetacean literature [1-10]. The epibiota reported from cetaceans consists of obligatory ectoparasites that are dependent on their hosts for survival in terms of nutrition or transport, and, of opportunistic commensals that attach onto marine hosts or flotsam and filter food particles from the marine plankton.

The abundance of epibiotic organisms on a host population is affected by a complex interaction of physical and biological factors and changes in the epibiota may serve as a biological indicator of ecosystem shifts that may be difficult to observe by other means. Shifts in the epibiota macrofauna on a particular host, therefore, may allude to larger and more complex environmental changes at play [11-16]. Changes in the epibiota of cetaceans may give indications of altered migration patterns in the host population or changed proportions of seasonally overlapping populations, which migrate into the study area from spatially separated grounds. Changes in abundance may also reflect changes in the parasite’s ecology in terms of fluctuations in the relative abundance of alternative hosts or changes in the physical environment that may affect their survival.

Little information exists on the epibiotic macrofauna on cetaceans in Icelandic waters. Sparse historical records have been obtained from sporadic observations and, to date, no systematic studies have been conducted. Of those that are known, Hallas [17] reported finding the caprellid *Cyamus boopis* Lütken, 1870 (Malacostraca: Cyamidae) and the cirripedes *Coronula diadema* (L.) (Maxillopoda: Coronulidae) and *Conchoderma auritum* (L.) (Maxillipoda: Lepadidae) from humpback whales, *Megaptera novaeangliae* Borowski, 1781, off the south coast of Iceland. The specimens of *C. auritum* were observed attached to the sessile barnacle *Coronula,* whilst a further single specimen was found attached directly to the skin. Later, Stephensen [18] reported *Pennella balaenoptera* Koren et Danielsson, 1877 (Copepoda: Pennellidae) from common minke whales, *Balaenoptera acutorostrata* (syn. *rostrata*) Lacépède, 1804, in Icelandic waters. *Cyamus ovalis* Roussel de Vauzème, 1834 was listed from a North Atlantic right whale, *Eubalaena glacialis* (Müller, 1776), in a catalogue of whale lice in the collections of the British Museum [19]. The last recorded species is the sea lamprey, *Petromyzon marinus* L. (Agnatha: Petromyzontidae), from photographs of killer whales, *Orchinus orca* L., in the waters south of Iceland [20].

Common minke whales are commonly observed in the waters off Iceland between April to November, although their abundance is at its peak in June and July [21]. The winter distribution of the population, however, is not fully established. While some individuals may overwinter in Icelandic waters, the bulk of the population is believed to migrate to southern locations as far south as West Africa [22].

The current study, which forms part of a larger co-ordinated programme of collaborative research on the ecology and biology of common minke whales, set out to provide baseline information on the species composition, geographic distribution and abundance of the epibiotic macrofauna on minke whales in Icelandic waters during the summer period. In the light of projected environmental changes in the world’s oceans in the coming decades, the information may prove valuable as a basis for future comparisons.

## Methods

**Figure 1:**
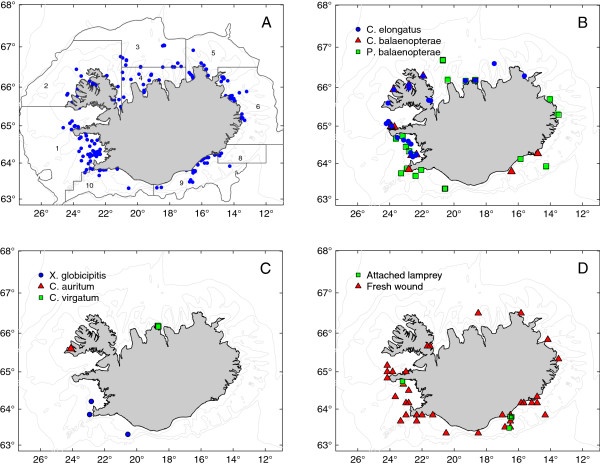
**Maps of Iceland showing the landing point of each common minke whale (*Balaenoptera acutorostrata *Lacépède, 1804) and details on their epibiotic macrofauna that was subsequently found.** (**A**)† all minke whale landings; **B**) Copepod ectoparasites: *Caligus elongatus* von Nordmann, 1832, *Cyamus balaenopterae* Barnard, 1931, and, *Pennella balaenopterae* Koren et Danielsson, 1877; **C**) Cirripede barnacles: *Conchoderma virgatum* Spengler, 1790, *C. auritum* L., and, *Xenobalanus globicipitis* Steenstrup, 1851; and, **D**) Live lampreys, *Petromyzon marinus* L., attached and whales bearing fresh lamprey scars. † Geographical area divisions follow definitions of oceanic areas around Iceland [see [24].

**Figure 2:**
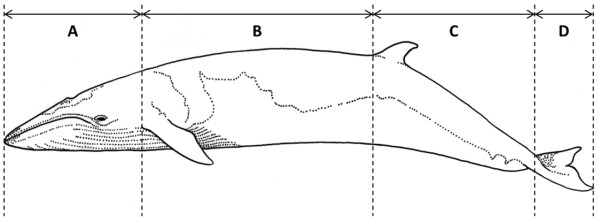
**Division of the common minke whale’s (*Balaenoptera acutorostrata *Lacépède, 1804) body into zones used for the epibiotic macrofauna study. A** = head; **B** = leading edge of the flippers to leading edge of the dorsal fin; **C** = dorsal fin to terminus of the peduncle; and, **D** = tail flukes.

**Figure 3:**
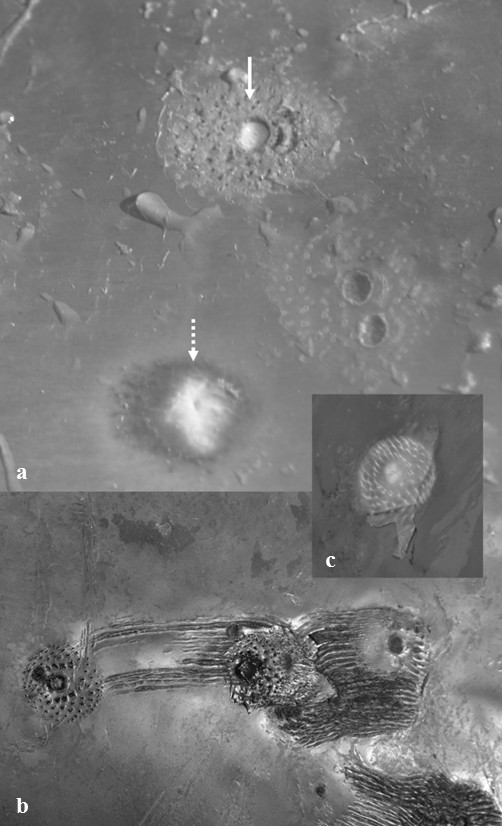
**Lamprey, *Petromyzon marinus *L., scars on the skin of common minke whales (*Balaenoptera acutorostrata *Lacépède, 1804)*. *a**) Recent (solid arrow) and old (dotted arrow) feeding wounds; **b**) dentition marks and scores left in the skin by an attached lamprey; **c**) dentition pattern following the removal of a live lamprey.

A study on ectoparasites, epizoics and sea lamprey marks on common minke whales was carried out in relation to a comprehensive research programme on the ecology and biology of common minke whales in Icelandic waters [23]. Epibiotic data and samples were collected from 185 animals and lamprey skin lesions examined on 188 animals landed between May to September 2003 to 2007 (Table 1, Figure 1A). The whales were taken on-board a vessel about half an hour to 5 hours post-mortem. The tail fluke and one lateral side of each whale were examined for epibiotic macro-organisms and skin lesions by eye immediately following the removal of the whales from the water. The intensity for each species in each of four body regions (Figure 2) was recorded and qualitative sub-samples were taken for subsequent identification in the laboratory. Lamprey scars were categorised as either “fresh” or “old” based on whether the attachment wounds on the epithelium were still open or had healed (Figure 3).

**Table 1:** **Spatial and temporal distribution of the common minke whales, *Balaenoptera acutorostrata *Lacépède, 1804, (n = 185) examined in the current study for their epibiotic macrofauna during their summer migrations in Icelandic waters over the period 2003 to 2007**

**Years**		**2007**	**2007**	**2004, 2006, 2007**	**2004, 2005, 2006, 2007**	**2003, 2005, 2006, 2007**	**2003, 2007**	
**Months**		**April**	**May**	**June**	**July**	**August**	**September**	**Total**
	1		3	8(+1) ^††^	11	17(+2)	7	46(+3)
	2	3	1	4	6	13	3	30
	3			7	5	2		14
	4			8	4	2	1	15
Geographic areas^†^	5			4	4	2	1	11
	6		2	7	6	6	2	23
	8				2	1		3
	9		1	2	14	8	2	27
	10			9	1	4	2	16
Total		3	7	49	53	55	18	185(+3)

†Geographic areas are detailed in Figure 3a.

†† Additional whales examined for lamprey scars only are shown in parentheses.

This study forms part of a comprehensive research programme on the ecology and biology of minke whales in Icelandic waters. The programme was granted a special permit to sample up to 200 minke whales by the government of Iceland according to article VIII of the Convention of the International Whaling Commission (IWC). All details of the survey described comply with the current laws of the Republic of Iceland.

Specimens of *Caligus elongatus* Nordmann, 1832 (Copepoda: Caligidae) and its monogenean hyperparasite, *Udonella caligorum* Johnston, 1835 (Udonellidae) were examined under an Olympus SZ30 dissecting microscope. Closer evaluation of a sub-sample of 50+ *C. elongatus* was performed using a compound Olympus BX51 microscope.

The prevalence, intensity and the mean intensity of each parasite burden was determined and follows the definitions provided by Bush *et al.*[25]. Where data was sufficient, the relationship between parasite intensity (*ln*) and the length of the whale was analysed using a least squares regression. Differences in prevalence of certain parasitic species between the two sexes and different time periods were investigated using chi-square tests.

## Results

From the examination of 185 common minke whales, three ectoparasites *Cyamus balaenopterae* Barnard, 1931 (Amphipoda: Cyamidae) and *C. elongatus* and its monogenean hyperparasite *U. caligorum* were found; one mesoparasite species, *P. balaenopterae* three epizoics, *Conchoderma virgatum* Spengler, 1790, *C. auritum* and, *Xenobalanus globicipitis* Steenstrup, 1851 (Cirripedia: Balanidae). On five occasions, a single live sea lamprey, *P. marinus* was found attached to a whale. In addition, fresh (*i.e.* open) and old feeding/attachment wounds, were commonly seen on the flanks of whales (see Table 2). All five *P. marinus-*infected minkes were landed in waters to the southwest and to the southeast of Iceland in July and August 2005 and 2006 (Table 2, Figure 1D). Four of the lampreys fell off the whales when they were hauled from the water and, therefore, it may be assumed that the prevalence of attached lampreys is underestimated in the present study with some specimens possibly detaching from the host unnoticed. Fresh lamprey scars were frequently observed on the posterior part of the flank of 20 out of 188 examined whales landed to the west and south of Iceland, suggesting recent associations between the two (P = 10.6%), (Table 2, Figure 1D). Old scars were observed on a further 85 minke whales from all areas (P = 45.2%).

**Table 2:** **Infection statistics for the ectoparasites and epizoics recovered from one lateral flank and the fluke of each common minke whale, *Balaenoptera acutorostrata *Lacépède, 1804, (n = 185) landed in Icelandic waters during the period April to September 2003-2007**

				**Regression of body length against *ln *(intensity)**		**Chi test prevalence on females *vs*. males (df = 1)**		**Chi test prevalence in April-July *vs*. Aug-Sep (df = 1)**	
	Habitat†	P (%)	M.I	r	p	*χ*2	p	*χ*2	P
**Phylum Arthropoda**									
**Fam. Caligidae**									
*Caligus elongatus*	all	11.9	95.5	0.23	0.32	1.45	0.23	28.88	<0.001
**Fam. Pennellidae**									
*Pennella balaenopterae*	B, C, D	10.3	1.6	-	-				
**Fam. Cyamidae**									
*Cyamus balaenopterae*	all	6.5	37.0	0.19	0.59	5.08	0.02	13.69	<0.001
**Fam. Lepadidae**									
*Conchoderma virgatum*	B, C (*)	0.5	4.0	-	-				
*Conchoderma auritum*	A (baleen plate)	0.5	1.0	-	-				
**Fam. Coronulidae**									
*Xenobalanus globicipitis*	D	1.6	5.3	-	-				
**Phylum Chordata**									
**Fam. Petromyzontidae**									
*Petromyzon marinus* attached	B, C	2.7	1.0	-	-				
*P. marinus* fresh scars	B, C	10.6	na						
*P. marinus* old scars	A, B, C	45.2	na						
**Class Monogenea**									
**Fam. Udonellidae**									
Udonella caligorum**	all	6.6	na						

Abbreviations: *M.I.*, mean intensity; p, probability; *P*, prevalence; *r*, regression coefficient; *χ*2, chi-squared; na, not available.

†For a description of the “*Habitat”* regions see Figure 1; * Attached to *P. balaenopterae*; ** Infection statistics on *C. elongatus* based on the microscopic evaluation of a sub-sample of 332 *C. elongatus*.

Twenty-two whales were found with a total of 2006 *C. elongatus* attached, representing the first time that this parasite has been reported from a cetacean (Table 2; Figure 1B). The *Caligus* specimens were found distributed over the entire exterior of the whale with no apparent preference for a particular habitat (P = 11.9%; M.I. = 95.5). No significant relationship between the intensity of *Caligus* and the length and/or sex of the whale was found. A significant seasonal trend in the prevalence of *C. elongatus*, however, was observed (*χ*2 = 28.88; p<0.001: d.f. = 1) (Table 2). The copepods were principally observed on minke whales landed in August and September; they were observed on only one of the whales landed in July and no infections were observed on minkes sampled in April to June. Closer evaluation of a sub-sample of *C. elongatus* revealed that there were at least two morphotypes of *C. elongatus* present, those where the swimming legs were separate and, a small number of sea lice, where the posterior swimming legs were fused. A full morphological and molecular study of both forms is in progress and will be presented elsewhere.

The exterior of *C. elongatus* was also infected with the eggs, juveniles and adults of a monogenean hyperparasite, *U. caligorum*. A total of 22 *U. caligorum* were found on a sub-sample of 332 *C. elongatus* that were examined (P = 6.6%), (Table 2).

*Cyamus balaenopterae* was found on all body regions of the minke whales landed off the west and south coasts (P = 6.5%; M.I. = 37.0), (Table 2, Figure 1B). Twelve whales were infected with *C. balaenoptera,* although a significantly larger proportion of the males were infected than were the females (*χ*2 = 5.08; p<0.05: d.f. = 1). Few lice were observed before August and a significant difference was observed in the prevalence of *C. balaenopterae* seen in the period April to July and those seen between August to September (*χ*2 = 13.69; p<0.001: d.f. = 1).

A single *C. auritum* was found attached to a baleen plate on a 7.9 m male minke whale from the northwest coast in August 2005 (Table 2, Figure 1C). A second, 5.3 m female, minke whale landed off the north coast in September 2003 was infected with a specimen of *P. balaenopterae* onto which four specimens of *C. virgatum* were attached. *Pennella balaenopterae* was found anchored into the flesh of 19 minke whales with a maximum intensity of 5 parasites observed on one host (P = 10.3%; M.I. = 1.6). The copepod was recorded in all months that whales were sampled and from all the study areas, no infections were found on the host’s head region (Table 2, Figure 1B). The barnacle *X. globicipitis* was found firmly attached to the tail flukes on three whales (P = 1.6%; M.I. = 5.3), landed off the south and southwest coasts in July and August in 2005 and 2006 (Table 2; Figure 1C).

## Discussion and conclusion

*Caligus elongatus* was found on 11.9% of the minke whales investigated in this study, principally from those landed off the west and north coasts of Iceland. Although *C. elongatus* has been recorded from a wide spectrum of fish hosts in temperate waters [26-29], the current finding of specimens on minke whales, is to the authors knowledge, the first time that these have been found on a cetacean host. Although *Caligus* sp. larvae have been observed on young cod, *Gadus morhua* L., in Icelandic waters [30], detailed information on their distribution on the fish species inhabiting Icelandic waters awaits further examination. *Caligus elongatus* adults, however, are good swimmers and occur in the plankton as well as attached to hosts [31,32]. Studies on pen-reared Atlantic salmon, *Salmo salar* L., and southern bluefin tuna, *Thunnus maccoyii* (Castelnau, 1872), have shown that adult *Caligus* on wild fish that are attracted to the sea cages transfer onto the cage held stock [33,34]. It is also probable, therefore, that free swimming adult *Caligus* attach to whales rather than infections establishing from larvae attaching to whales. A parallel analysis of the stomach contents of individual whales sampled in the current study, revealed the frequent occurrence of *Caligus* sp. (Víkingsson *pers. comm*.), suggesting that common minke whales may become infected when filter feeding on infected fish. Although some specimens of *C. elongatus* were observed to have material within their guts, suggesting they had recently fed, the source of this material is not known. Stable isotope or molecular studies on the gut contents of *Caligus* on whales may help answer whether the lice actively feed on the whale [34].

The prevalence and mean intensity of *C. elongatus* on wild fish in southern Norway have been shown to increase from spring to autumn as a result of faster development in the warm summer months and formation of multiple generations throughout the summer [35,36]. This is in line with the higher prevalence of *C. elongatus* observed on the minke whales landed late in the summer in the present study, suggesting the local origin of the infections and real seasonal shifts rather than inter-annual fluctuations due to different sampling distribution between years (see Table 1).

*Cyamus balaenopterae* was found on all body regions on the minke whales caught off the west and south coasts in the present study. The species is an obligatory parasite foraging on the whale’s skin [37] and is found globally on baleen whales [2,13,19,38-40]. Studies on *Cyamus scammoni* Dall, 1872, a related species found on the gray whale, *Eschrichtius robustus* Lilljeborg, 1861, revealed that they have a one year long direct life-cycle [41]. The larvae hatch from eggs in autumn, with the young remaining in the female’s brood pouch for two to three months. The juveniles are released from the pouch in mid-winter and attach to the soft skin on the belly or shield themselves from the water current by lying in scars on the host’s surface or in the orifices of the cirriped *Cryptolepas rachianecti* Dall, 1872. Most of the lice observed in Leung’s study had reached maturity in March and possessed a full brood by the time the whales arrived at the summer grounds. The life-cycles of two other *Cyamus* species, C. kessleri Brandt, 1872 and C. ceti (L., 1758), parasitising gray whales, displayed similar life-cycle patterns [41].

The low prevalence of *C. balaenoptera* observed in the early summer months in the present study may suggest that either the appearance and development of *C. balaenopterae,* in Icelandic waters*,* is later than that of *C. scammoni* or that given the smaller size of *C. balaenopterae* in the early summer, they were overlooked, sheltering within pores on the whale, which are preferred sites among Cyamidae species [42].

A tentative explanation of the significantly larger proportion of male minke whales infected with *C. balaenoptera* than females in the present study, may be due to the different migration routes taken and the geographic segregation of the sexes during the potential period of infection.

Studies of the Antarctic minke whale, *Balaenoptera bonaerensis* Burmeister, 1867, revealed a positive relationship between the occurrence of *C. balaenopterae* and the number of corpora in the ovaries of adult females [14]. This may be interpreted as increased abundance with larger host size. In the present study, however, there was no significant relationship between lice intensity and whale body length.

*Pennella balaenopterae* has a global distribution and is reported on a wide range of cetacean species [2,5,8,13]. It is the only *Pennella* species parasitising cetaceans, whereas other species of the genus are found embedded in the flesh of a wide range of marine hosts [43]. The life-cycle *P. balaenopterae* is poorly understood and only the adult female and the first naupliar stage have been identified with certainty [43,44]. Observations of the copepod in all months, years and areas of the present study show that the parasite can survive in the colder waters and contradicts Mackintosh and Wheeler [4] suggesting that the parasite falls off their host during migrations into colder waters.

A single *C. auritum* was found attached to the baleen plate of a minke whale landed on the northwest coast in August. This cirriped species is commonly found attached to ships and floating objects in tropical and warm temperate waters indicating that the settlement on whales moving into Icelandic waters occurs during winter migrations at lower latitudes. The higher prevalence of *C. auritum* observed on female, rather than male, sperm whales in the south Pacific further indicates that they are picked up in warmer water. Part of the population of male sperm whales migrate to higher latitudes in the Atlantic and Pacific Oceans, whereas the females remain in waters below a latitude of 40-45° all year around [45]. *Conchoderma auritum* rarely attaches directly to the skin of cetaceans and is mainly reported to be epizoic on hard surfaces including sessile *Coronula* barnacles, and occasionally on the teeth and baleen plates of whales [6,46]. Most *C. auritum*, therefore, are reported from humpback whales carrying settlements of *Coronula* spp. Other baleen whales appear to be rare hosts for *C. auritum* and there is only one previous record of this barnacle attaching to a minke whale, where a cluster of *C. auritum* was observed attached to the damaged baleen plates of a minke whale caught off the coast of East Greenland in 1984 [46]. The study, however, found no *C. auritum* infection on the 1317 minke whales that were examined from the North Atlantic over the period 1972 to 1984, further emphasizing the rarity of these incidences. In addition, very low prevalences of *C. auritum* on blue, *Balaenoptera musculus* (L.), fin, *Balaenoptera physalus* (L.), and sei, *Balaenoptera borealis* Lesson, 1828, whales have been reported [see [6].

In the current study, four *C. virgatum* were found attached to a single *Pennella* on a minke whale off the north coast. The barnacle is found attached to flotsam and on ships, as a hyperepizoic on *Pennella* or on the stalked barnacle *C. auritum* and has been reported from several large whale species, including minke whales [6]. The distribution of *C. virgatum* is circum-global in tropical and subtropical waters and its occurrence on whales in colder areas may be explained by migration from warmer seas. The finding of *C. virgatum* on whales late in the feeding season in the colder waters off the north coast of Iceland in the present study suggests that the barnacle may survive in the colder waters. The completion of its life-cycle though is most likely restricted to warmer areas.

The barnacle *X. globicipitis* has been reported from a number of cetacean species inhabiting tropical to temperate waters [47-49]. This barnacle species is typically found attached to the trailing edge of cetacean flukes and fins; its morphology is well adapted to the strong currents generated by the swimming movements of its host [50,51]. The average swimming speed of the host does not appear to be a factor affecting barnacle settlement, whereas diving to great depths may reduce settlement of the larvae [49].

Attempts have been made to use *X. globicipitis* as a biological tag to trace migration routes and the delineation of host populations. Spatial differences in the prevalence of *X. globicipitis* on Antarctic minke whales examined in summer, suggests that these whales also have separate winter grounds where the exposure to the barnacle is different [14]. A study on Mediterranean striped dolphins, *Stenella coeruleoalba* (Meyen, 1833), concluded that an increased prevalence of *X. globicipitis* on certain individuals was due a viral epizootic in the population predisposing individuals to infection [16,52]. Previous records of the species from Greenland and Finnmark in northern Norway [47] together with the present study probably represent the northern limits of their distribution in the North Atlantic. These northern records were all from balaenopterid hosts that most likely carried the barnacle from winter grounds at lower altitudes.

Kane *et al.*[49] commented that ocean productivity and therefore the availability of food for filter feeding barnacle species like *X. globicipitis* may cause spatial variation in their distribution. More information on the host selection criteria, environmental tolerance limits and early life history strategies is needed to determine the utility of *X. globicipitis* as a biological tag for cetaceans.

*Petromyzon marinus* is the only lamprey species reported in Icelandic waters [53]. It attaches to the surface of its host using an array of small keratinous teeth and rasps holes through the skin using its tongue, creating open lesions that leave pale scars when they heal [54]. Both the fresh and old scars seen on the minke skin, therefore, can be attributed to the activity of this one lamprey species. The force of the lamprey’s attachment to its host, however, is not strong and it may slip over its host’s surface. A single lamprey, therefore, may be responsible for inflicting numerous scars on a single host preventing an accurate estimation of intensity based on the observation of scars. The distribution of *P. marinus* is limited to temperate waters in the North Atlantic and until recently, findings of this species in Icelandic waters were rare [53]. An unusual abundance of free swimming lampreys, however, were noted in southwest Iceland in 2004 [55] and the number of lesions on salmonid fish south Icelandic rivers have increased in recent years [53]. A photo-identification based study on killer whales, *Orcinus orca* L., inhabiting the waters around the Vestmannaeyjar archipelago to the south of Iceland in July 2009, found two *P. marinus* attached to whales and the presence of shallow marks suggested other attachment events [20]. Re-sightings of the same individuals showed, evidently, that the attachments were of local origin and that the shallow marks were not permanent. Working through the photo-ID catalogue of whales in the area dating back to 1980, revealed no indication of lamprey marks in previous years. The finding of lampreys on minke whales, in the current study, relatively late in the summer further suggests that *P. marinus* can survive in the area, at least in the warmer waters off the south and southwest coasts.

The apparent change in the distribution of recent years is presumably related to rising sea temperatures [56], however, there is no evidence to suggest that lampreys complete their life-cycle in Iceland by spawning in Icelandic freshwaters [57].

Mackintosh and Wheeler [4] assumed that epizoic barnacles and *Pennella* fell off their hosts as they migrated into colder waters. The low prevalence of these epizoics observed in the present study prevents any firm conclusions being drawn. *Pennella balaenopterae* was found on minkes that were landed in each month, although the barnacles were found relatively late in the season; *C. virgatum* in mid-September, *C. auritum* in August, and, *X. globicipitis* in late July and August. This shows that at least some epizoic barnacles stay attached on the whale host throughout the feeding season in Icelandic waters. Lack of information on the epizoic species found on minkes from the winter grounds prevents a comparison with those found on whales in the summer grounds and, therefore, a concluding statement on whether the observed low prevalences in Icelandic waters are due to unfavourable conditions that are fatal to the barnacles.

The long term impact of increased sea temperatures as observed in the world’s oceans recent years and the projected subsequent rise in the near future [58] on the biota in the northern North Atlantic is an unknown. Potential future changes in the epibiotic macrofauna on common minke whales may indicate the altered migration route of the host population or alterations to the survival rate of the epizoic species.

## Competing interests

The authors declare that they have no competing interests.

## Authors’ contributions

DO organised the sample collections, analysed the data and wrote the initial draft. APS subsequently identified the *Caligus elongatus* and *Udonella caligorum* specimens, intellectually supported the study and corrected the manuscript drafts. Both authors read and approved the final manuscript.
